# Miscellaneous Notices

**Published:** 1851-10-01

**Authors:** 


### Miscellaneous Notices.

*On the Health of Women at the Critical Periods of Life.* By E. J. Tilt, M.D., &c., &c. London. 1851.

This will prove to be a very useful little work. It is elegantly written, and contains much useful and valuable information. If the suggestions offered by its amiable author are acted upon, what an amount of human suffering will be prevented. We quote a passage having reference to the prevalence of cerebral symptoms in the diseases of women, and on the exhibition of sedatives for their cure and alleviation.

"During the last thirty years there has been too much timidity in the employment of sedatives, and particularly in that of opium; for the present generation of medical men seem to forget, that often nature only requires to be freed from present pain, to enable an organ to return at once to the regular performance of its function, without future distress or inconvenience. Is it then necessary to remind the reader, how generally useful sedatives are, that in most diseases they not only assuage the acuteness of pain, but lull excited action to a slower rate of progress, and to a more subdued tone? The bloodvessels serve under the immediate rule of the accompanying nerves; and hæmorrhage as often depends upon their perturbed agency as on any peculiar state of the bloodvessels themselves; and although other measures may be indispensable, the return of such hæmorrhages can only be prevented by a judicious use of sedatives.

"We have already seen how useful is opium in the relief of many deranged states of action, particularly in dysmenorrhœa, and the utility of sedatives in diseases of the change of life might be deduced from the great frequency of cerebro-spinal symptoms at the cessation of menstruation. It will be remembered that I stated their frequency to be—

CEREBRAL SYMPTOMS.

| | | | |
|---|---|---|---|
| Headache, sick-headache, hysteria, and pseudo-narcotism had existed in | | 64 | per cent. |
| They were augmented in | 36 | | " |
| " remained the same in | 18 | | " |
| " were less in | 10 | | " |
| They did not exist in | | 36 | " |
| | | 100 | |

SPINAL SYMPTOMS.

| | | | |
|---|---|---|---|
| Spinal or dorsal pains had existed in | | 70 | per cent. |
| They were augmented in | 46 | | " |
| " the same in | 17 | | " |
| " less in | 7 | | " |
| They did not exist in | | 30 | " |
| | | 100 | |

| | | | |
|---|---|---|---|
| Hypogastric pains of a bearing-down character, referred by women to the womb and ovaries, had existed in | | 51 | per cent. |
| They were augmented in | 30 | | " |
| " the same in | 12 | | " |
| " less in | 9 | | " |
| There were none in | | 49 | " |
| | | 100 | |

"This frequency of cerebro-spinal symptoms warrants an equally frequent exhibition of sedatives, and I do not hesitate to say that, under some form or other, they are always required in diseases of cessation. In the milder forms of catamenial headache and pseudo-narcotism they alone suffice to cure, and they always assist the action of bleeding, of purgatives, and of other remedies which may be deemed necessary."

*Miss Martineau and her Master.* By J. STEVENSON BUSHNAN, M.D., &c. 1 vol. 8vo. London. 1851.

AN able, searching, and philosophical *exposé* of Miss Martineau and Mr. Atkinson's trashy volumes. Having in our last journal so fully analyzed the work which Dr. Bushnan has so unmercifully mauled, we have not the face again to trespass upon the patience of our readers by going into the subject. We think Dr. Bushnan has magnified this pair of pseudo-philosophers into too much importance by devoting *a volume* to the consideration of their inanity.* The little work before us is forcibly written, and exhibits, on the part of its author, analytical powers of a high order.

*On Puerperal Insanity.* By F. W. MACKENZIE, M.D., &c. London, 1851. Pamphlet. pp. 22.

THIS essay was originally published in the "London Journal of Medicine." We are much pleased with it. The author is evidently an observing, experienced, and intelligent man. His style is perspicuous, and nothing can be better than his mode of relating a case. In consequence of our not receiving this pamphlet until our Journal was nearly printed, we have been deprived of the pleasure of presenting to our readers an analysis of its contents. We hope to have another opportunity of reverting to it.

*A Voyage to China, &c., &c.* By DR. BERNCASTLE. 2 vols. 8vo. With Illustrations. London. W. Shoberl. 1851.

THIS is one of the most recent and interesting works on the Celestial Empire, since the publication of Dr. Downing's "Fanqui in China." It is full of information to the professional as well as the general reader. These volumes should be read by every one who takes an interest in the progress of civilization in the East.

